# Optimization, characterization and biological activity of siderophore produced by marine *Streptomyces coelicolor*

**DOI:** 10.1371/journal.pone.0341555

**Published:** 2026-02-02

**Authors:** Benazir Begum Sirajudeen, Jenifer Annis Christy Joseph Sahayaraj, Lok Kirubahar Sankararamalingam, Raja Roobia Rajendran, Murugappan Ramanathan, Padmavathy Sethuramasamy

**Affiliations:** 1 PG and Research Department of Zoology, V. O. Chidambaram College, Tuticorin, Tamil Nadu, India; 2 Department of Biotechnology, Mary Matha College of Arts and Science, Periyakulam, Tamil Nadu, India; 3 Department of PG Zoology, Aditanar College of Arts and Science, Tiruchendur, Tamil Nadu, India; 4 Department of Zoology, Thiagarajar College, Madurai, Tamil Nadu, India; 5 Department of Microbiology, Thiagarajar College, Madurai, Tamil Nadu, India; Friedrich Schiller University, GERMANY

## Abstract

Two *Streptomyces* *spp.* were isolated from open ocean waters of the Gulf of Mannar. Of the two isolates, one was identified as *Streptomyces coelicolor* through conventional and molecular approaches, and it was found to produce a siderophore. Characterization revealed the siderophore to be of trihydroxamate type with hexadentate iron-binding capacity. FTIR analysis indicated the presence of aromatic rings with C–O and C = C stretching, while ^1^H and ^13^C NMR together with mass spectrometry confirmed the hydroxamate nature and identified the siderophore as ferrioxamine. A narrow shift in λ max indicate the photoreactive nature of the siderophore on exposure to sunlight. The cell-free supernatant of *S. coelicolor* and the purified siderophore dose-dependently inhibited the growth of microbial pathogens. Quorum quenching activity was confirmed using the indicator strain *Chromobacterium violaceum*. Both the cell-free supernatant and siderophore were found to inhibit biofilm formation and induce reactive oxygen species (ROS) generation in pathogens. The siderophore also suppressed the proliferation of the breast cancer cell line (MCF-7) by disturbing iron homeostasis. Optimization of International Streptomyces Project (ISP2) medium constituents using two-level factorial design and response surface methodology (RSM) enabled cost-effective siderophore production. In addition to iron, the siderophore exhibited binding affinity for other heavy metals including zinc, cobalt, cadmium, lead, and magnesium.

## Introduction

Siderophores are low-molecular-weight (<10kDa), multidentate, iron-chelating organic compounds produced by most of the microorganisms. Iron is an indispensable element for various cellular activities. Iron functions as a cofactor for enzymes involved in regulating cell growth and proliferation. Siderophore mediated iron sequestration was reported to inhibit cell growth by influencing various biological and metabolic processes. To meet the iron requirement, cancer cells perturb their iron metabolism for plentiful supply of iron to sustain rapid proliferation, increased invasion, and metastasis [1]. In line with the above, apoptosis can be triggered in the tumour cells by iron chelation [2]. Desferoxamine a commercially available synthetic siderophore was reported to exhibit anti-tumor properties by limiting iron bioavailability to malignant cells [3].

Apart from inducing apoptosis in the cancerous cells, siderophore exhibits wide range of inhibitory efficiency against pathogenic microbes [4]. Renaissance towards the discovery of novel secondary metabolites from diverse microbial sources is in limelight in the recent years due to the development of resistance development in pathogenic microbes towards commercially available antibiotics [5].

Biofilm formation plays a crucial role in determining the toxicity of pathogenic microbes as well as in the contamination of medical implants and other equipment [6]*.* The synthesis and expression of virulence factors is regulated by cell-to-cell chemical communication using quorum-sensing molecules namely, N-acyl-homoserine lactones (AHLs) [7]. Biofilm formation and virulence factor expression in microbes is regulated by quorum sensing. The disruption of the quorum sensing network is a viable approach to inhibit the biofilm formation in pathogenic bacteria [8].

In addition to iron chelation, siderophores play a crucial role in the biogeochemical cycling and biological uptake of various metals such as aluminum (Al), cadmium (Cd), copper (Cu), manganese (Mn), lead (Pb), and zinc (Zn), as well as trace amounts of radionuclides like gallium-67 and indium [9]. Therefore, siderophore-mediated sequestering system is reported to mitigate heavy metal toxicity in various environments [10]. Since siderophores have a wider range of applications, their production has to be enhanced under *in vitro* conditions by the optimization of constituents in the growth medium.

Actinomycetes are an important source of secondary metabolites, accounting for two-third of microbial bioactive metabolites discovered so far [11]. The majority of the secondary metabolites with pharmacological application are produced by the members of the genus *Streptomyces*, the highly represented member of actinobacterial group [12]. Consequently, *Streptomyces sp*., from marine environment are reported to be the largest and an outstanding repository of myriads of bioactive compounds with diverse chemical structures [13]. Siderophores are one such bioactive secondary metabolites. More than 500 different siderophores were reported, of which 270 are well characterized [14], while the rest remain uncharacterized, and their functions are yet to be determined [15]. Therefore, knowledge on siderophores produced by marine Streptomyces is relatively new and only a few reports are available.

In consideration of all the above, in the present study, siderophore producing *Streptomyces spp.* were isolated from the open ocean water collected from Gulf of Mannar and identified by molecular methods. Siderophore produced by the isolate was characterized by FT-IR, GC-MS, ^1^H NMR and ^13^C NMR spectra analyses. Siderophore production by the isolate was enhanced under *in vitro* conditions using a laboratory scale bioreactor by optimizing the constituents of the growth medium (ISP2) by Response Surface Methodology-Central Composite Design (RSM-CCD). Metal chelation efficiency, antioxidant potential, biofilm inhibition, quorum quenching and antimicrobial efficiency of the siderophore were analysed.

## Materials and methods

### Sample collection and isolation of Streptomyces

Open ocean surface water samples were collected at three different locations about one nautical mile away from the seashore of the village Vethalai (latitude 9°15’51.8“N and longitude 79°06’25.0”E) at Gulf of Mannar, Tamil Nadu, India. Aseptic conditions were followed during the collection and transportation. The samples were incubated in a water bath at 50°C for 10 min to reduce non-sporulating bacteria [16]. After heat treatment, the samples were serially diluted (10^-1^ to 10^-6^) with sterile sea water. From each dilution, 0.1mL was evenly spread onto the International Streptomyces Project (ISP2) agar medium supplemented with cycloheximide (50 µg/mL), nystatin (40 µg/mL) and nalidixic acid (50 µg/mL). Five replicates were maintained for each dilution and the plates were incubated at 30°C for 10 days. After incubation, colonies displaying characteristic *Streptomyces* morphology, such as a tough, leathery texture; chalky or powdery aerial mycelium; and distinctive pigmentation were carefully selected. Selection criteria were based on standard descriptions of *Streptomyces spp*. in Bergey’s Manual of Systematic Bacteriology. Colonies selected were then streaked onto fresh ISP2 medium to obtain pure cultures. The pure cultures were stored on sterile vials containing 20% glycerol solution at −20°C for further analysis.

### Screening and extraction of siderophores

To elucidate siderophore production, the isolates were grown in iron-depleted minimal medium (MM9) containing 0.3 g l-1 KH_2_PO_4_, 0.5 g l-1 NaCl, 1.0 g l-1 NH_4_Cl, 6.0 g l-1 NaOH and 30.24 g l-1 piperazine-N, N’-bis (2-ethanesulphonic acid) (PIPES). The medium was autoclaved and supplemented with 30 ml of 10% (m/v) deferrated casamino acids (iron was removed with 3% 8-hydroxyquinoline in chloroform), 2.0 g l-1 glucose, 1 ml 1M MgCl_2_ and 1 ml 100 mM CaCl_2_ [17]. After 7 days of incubation at 30°C (with shaking −120 rpm), the medium was centrifuged at 10,000 rpm for 15 min and the cell-free supernatant was examined for presence of siderophore by FeCl_3_ test, chrome azurol sulphonate (CAS) assay and CAS agar plate test [18]. The nature of the siderophore produced by the isolates was ascertained by examining absorption maxima (kmax) of ferric–siderophore complex. Furthermore, the supernatant was subjected to Csaky, Arnow, and Vogel’s assay [19] to confirm the nature of siderophore produced.

### Characterization of *Streptomyces sp.*

*Streptomyces* strain that produces siderophore was identified by morphological, physiological, biochemical and molecular tests. Morphological characters such as shape of the colonies, colour of the mycelium and production of diffusible pigments were observed. Isolates were subjected to various biochemical tests according to Bergey’s Manual of Determinative Bacteriology [20] and identified based on the procedure described in international Streptomyces project (ISP) [21]. In addition to conventional biochemical analyses selected isolates were subjected to 16S rRNA gene partial sequencing and phylogenetic analysis.

Genomic DNA of *Streptomyces sp.* that produce siderophore was extracted using Qiagen DNA isolation kit. The 16S rRNA gene sequence of the isolate was amplified using the universal primers 785F 5`-GGATTAGATACCCTGGTA-3` and 907R 5`-CCGTCAATTCMTTTRAGTTT-3` and compared with related gene sequences available at the National Center for Biotechnology Information (NCBI) database. Evolutionary history of the strain was inferred using neighbour-joining dendrogram using the Molecular Evolutionary Genetics Analysis software package (MEGA version 4.0). The topology of the phylogentic tree was evaluated by the bootstrap resampling method [22].

### Purification of siderophore

Siderophore produced by the isolate was purified using Amberlite XAD 400 resin and Sephadex LH-20 column as described by Sayyed and Chincholkar [23], lyophilized and stored at −20°C for further structural analyses. Purification of siderophore produced by the isolate involves several steps, initially the isolate was cultured in a 3L bioreactor (Lark Scientific, India) containing 2L of ISP2 medium at 30°C ± 1°C and 120 rpm for 10 days. The broth culture was centrifuged (15,000 rpm for 15 min) at 4°C. The supernatant was filtered (0.2 μm) and acidified to pH 3.0 using concentrated HCl and passed through a column (5X30) packed with Amberlite XAD-400. Siderophore was eluted from the column using 50% methanol. Eluted fractions were checked for the presence of siderophore by CAS test. CAS positive fractions were concentrated on a rotary vacuum evaporator. The XAD concentrated fraction of siderophore was further purified using Sephadex LH-20 column and eluted with methanol. Subsequently, Thin layer chromatography (TLC) was performed to test the purity of the collected fractions with siderophore activity with a mobile phase containing methanol: ammonium acetate (60:40). Fraction with siderophore activity was freeze dried and further characterized to confirm the identity by performing spectroscopic assays.

### Characterization of the Siderophore

#### Fourier transform infrared spectroscopy (FT-IR).

Siderophore after lyophilization was pelleted with potassium bromide (KBr) and subjected to FT-IR spectroscopy (Jasco 460 plus) for determination of functional groups. Spectra were recorded in the range from 4,000–400 cm^-1^ and the results were averaged over 100 scans.

#### ^13^C and ^1^H Nuclear magnetic resonance (NMR).

^1^H and ^13^C NMR spectra of the lyophilized siderophore were recorded on BRUKER 500-MHz spectrophotometer equipped with triplet resonance probe and triple axis gradients. Lyophilized siderophore samples were dissolved in deuterated dimethyl sulphoxide (DMSO) and injected through a 5 mm one-dimensional NMR tube to record the nuclear magnetic resonance imaging with solvent signal as internal reference [24].

^13^C NMR is much less sensitive than ^1^H NMR, because of the low natural abundance of ^13^C (1.07% compared with 99.99% for ^1^H) and the significantly lower gyromagnetic ratio (6.7283 Å ~ 107 rad T-1 S-1) by about a factor 4. The high natural abundance (99.98%) and the high gyromagnetic ratio (26.7522 Å ~ 107 rad T-1S-1) of ^1^H, makes proton NMR as one of the most sensitive techniques.

#### Gas chromatography mass spectroscopy (GC-MS).

Molecular weight of the purified siderophore sample was determined using mass spectroscopic analysis. Mass spectrum data was obtained using a MAT 8230 ST (Finnigan MAT, Bremen, Germany) instrument equipped with auto sampler. Purified siderophore was dissolved in water/methanol/trifluoroacetic acid 50:50:0.1 (v/v/v). The sample (1 μL) was injected into the port. The ion of interest was selected then collided with helium gas to obtain fragments that was analyzed based on m/z. Helium with a purity of 99.99% was used as the carrier gas at a flow rate of 2.25 mL/min. Scanned data was obtained in a mass range of 100–1000 m/z and detector voltage was fixed at 1.8 Kv. [25].

#### Determination of hydroxamate nature.

Mono-, di- or trihydroxamate nature of the hydroxamate siderophores was ascertained by spectrophotometric method and paper electrophoretic method [26]. Shift in λ max as a function of pH value was used to distinguish the ferric complexes of hydroxamate siderophores. The paper electrophoretic method was performed to further distinguish the ferric complexes. The electrophoresis was run at 30 V cm^-1^ for 1–2 h with 4% formic acid (pH 2). The paper was dried to remove traces of formic acid and sprayed on both sides with 2% FeCl_3_ solution.

#### Ligand denticity of hydroxamate siderophore.

Hydroxamate siderophores are further distinguished into bi-, tetra- and hexadentate based on the number of bonds that the ligand forms with the metal ion. Ligand denticity of the hydroxamate siderophores was determined by their change in colour at different pH values (4–9). Ferric hexadentate type maintains a stable (red) colour over a wide range of pH. Bidentate and tetradentate hydroxamate siderophores exhibit a colour shift with varying pH [27].

#### Screening of siderophore metal complexation.

In addition to Fe^2+^, complexation of siderophore with other divalent metal ions was determined by CAS plate assay [18] and microtiter plate assay [28]. Petri plates containing different metals-CAS-HDTMA dye agar were prepared as described earlier. The following metals [ZnCl_2_, CuCl_2_.5H_2_O, CoCl_2_.6H_2_O, FeCl_3_.6H_2_O, CdCl_2_, Al_2_(SO_4_)_3_, Pb (NO_3_)_2_ and MnCl_2_.4H_2_O] were used during CAS agar plate preparation. The concentrations of all the metals were kept constant to 50 ppm. Cell free supernatant (200 μL) of the isolates was added to the well (8 mm) on the CAS plate and incubated at room temperature to observe the colour change.

For microtiter plate method, CAS-metal-HDTMA (hexadecyltrimethylammonium bromide) dye complex in equal proportion (120 μL) was dispensed to each well of a U-bottomed microtiter plate. Subsequently, 100 μL of cell-free supernatant was introduced into each well and gently mixed. Complexation of siderophore to the metal was determined based on the change in the colour of the dye. Supernatant of the uninoculated media served as a positive control. Cell free supernatant of *Lactobacillus sp*. was used as a negative control, since they lack siderophore synthesis.

### Photolytic properties

Siderophore produced by the isolate was dissolved in 0.2-µm-filtered, ultraviolet (UV)- irradiated seawater (pH 8) to a concentration of 10 µM. Trace metals and trace-metal-binding organic compounds from the seawater were removed as described by Macrellis et al. [29]. The solution was placed in an acid-washed quartz flask and exposed to natural sunlight for 6 h between 10:00 and 16:00 h. Identical solutions shielded from sunlight served as dark control.

### *In vitro* antagonistic activity

Antagonistic activity of the cell free culture supernatant of *Streptomyces sp.* and the purified siderophore were tested against six different bacterial strains viz., *Staphylococcus aureus* (MTCC 2940), *Pseudomonas aerugino*sa (MTCC 424), *Bacillus subtilis* (MTCC 2414), *Micrococcus luteus* (MTCC 106), *Salmonella typhi* (MTCC 734) and *Escherichia coli* (MTCC 46), and six fungal strains viz., *Aspergillus niger* (MTCC 281), *Aspergillus flavus* (MTCC 2206), *Penicillium oxalicum* (MTCC 4931), *Fusarium oxysporum* (MTCC 284), *Alterneria alternata* (MTCC 1362), and *Botrytis cinerea* (MTCC 359) by micro broth dilution elaborated by Cappuccino and Sherman [30] with slight modifications. The bacterial and fungal strains were obtained from the Microbial Type Culture Collection (MTCC), Chandigarh, India. The strains were selected based on their clinical relevance, pathogenic diversity, and representation of both Gram-positive and Gram-negative bacteria as well as filamentous fungi of medical and agricultural importance. For the microbroth dilution method, 20 µL of the inoculum (mid-log phase) was added to 180 µL of Muller Hinton broth supplemented with different concentration of cell free supernatant and purified siderophore to the wells of a flat-bottom transparent 96-well plate. The plates were incubated for 24 h (bacteria) and 48 h (fungi) at 37°C with shaking at 200 rpm. Absorbance was read at 600 nm after incubation using BioTek Epoch 2 Microplate Spectrophotometer. All experiments were conducted in triplicates. Dose-response influence of cell free supernatant and purified siderophores on different pathogenic strains was calculated using Prism software (ver. 6.07).

### Antioxidant assay

Free radical scavenging activity (RSA) of the siderophore produced by *Streptomyces sp.* was assessed by DPPH (1,1-diphenyl 1–2-picryl-hydrazyl) assay [31]. Briefly, one ml of different concentrations of siderophore was added to 3 ml of the DPPH solution (100 µM) followed by 30 min of incubation in the dark and absorbance was measured at the 517 nm in a UV-visible spectrophotometer. Gallic acid was used as a reference and methanolic DPPH was used as a positive control. The percentage RSA was calculated by using the following formula.


$$\%\;\text{RSA}\;=\;[({\text{A}}_{\text{0}}\;-\;{\text{A}}_{\text{1}})\;/\;{\text{A}}_{\text{0}}\times \;\text{100}$$

A_0_ is the absorbance of the control (without the sample), and A_1_ is the absorbance of the sample.

The inhibition concentration at which absorbance is reducing by 50% (IC_50_) were calculated by using average RSA.

### Anticancer activity

Cytotoxicity of siderophores produced by the isolates against human breast cancer (MCF-7) line was determined under *in vitro* conditions by colorimetric assay using Methyl thiazole tetrazolium (MTT) following the procedure of Chandrasekaran et al. [32]. Nontoxic nature of siderophores towards normal cells was determined using Vero cell lines. The breast cancer cell line (MCF-7) and normal cell (Vero) line were obtained from National Centre for Cell Sciences (NCCS), University of Pune, Maharashtra For cytotoxicity assay, MCF-7 and Vero cells were seeded separately on 96-well plates in 100 μL of medium containing 5% FBS, at plating density 10,000 cells/well and incubated for 48 h at 37°C, 5% CO_2_, 95% air with relative humidity (100%). Different concentrations of siderophores (100–1000 μL) were added to the wells and the medium without siderophores served as control. Experiments were carried out in triplicates for comparison. After 48 h of treatment, 50 μL of MTT (5 mg/mL) was added to each well and incubated for 4 h at 37°C. The medium with MTT was then flicked off and the formed formazan crystals were solubilized in 100 μL of DMSO and the absorbance at 570 nm was measured using a microplate reader. Cytotoxic effect of the commercially available siderophore desferrioxamine (Sigma, USA) was also determined.

The percentage of cell inhibition was determined using the following formula,


$$\%\;Inhibition=\text{1}\text{0}\text{0}-\left(\frac{OD\;Sample}{OD\;Control}\right)\;\times \text{1}\text{0}\text{0}$$

### Screening for quorum quenching activity

Quorum quenching activity of the siderophore and cell free supernatant was tested against the reporter/biosensor strain *C. violaceum* MTCC 2656 (Microbial Type Culture Collection) by microtiter plate method. Sterile nutrient broth (100 μL) along with equal volume of *C. violaceum* broth culture was dispensed in a U-bottomed microtiter plate well along with 20 μL of different concentration of siderophore and cell free supernatant. A positive and negative control was maintained. Experiments were carried out in triplicates. The titter plate was incubated at 30°C for 18 h and observed for violacein inhibition by measuring the OD at 595 nm in microplate reader.

### Biofilm inhibition assay

Biofilm inhibition potential of siderophore produced by the isolate was determined on 96 well flat bottom polystyrene micro-titer plates using crystal violet (CV) dye as described by Elshaer and Shaaban [33]. Cell suspension (10 μl) of *Staphylococcus aureus* having 0.5 O.D at 600 nm was inoculated in 190 μl TSB medium in each well and 20 μl of different concentrations of siderophore was added to the wells. A positive control (without inoculation) and negative control (with inoculation) was maintained. The plates were covered and incubated at 37°C for 24h. After incubation, the liquid and non-adhered cells were then removed from the wells by gently overturning the plate onto paper towels. Each well was then washed with phosphate-buffered saline (PBS) three times to remove the planktonic cells. After aspiration of planktonic cells, biofilms were fixed with 200 µl of 99% methanol and air dried.

Then, 200 μl of crystal violet solution (0.2%) was added to all wells and incubated for 10 minutes at room temperature. The excess dye was removed as before, by overturning the plate onto paper towels and washed twice with ddH_2_0 and plates were left to dry overnight under aseptic conditions. Finally, the cell bound CV was eluted using 205 μL of elution buffer (ddH_2_O with 40 mM HCl and 1/3 volume ethanol added). 80 μL of eluted CV was removed from each well and placed into wells of a new 96-well plate for measuring the optical density (OD) at 595 nm in microplate reader providing a quantitative measure of biofilm inhibition.

Experiments were carried out in triplicates, P-values comparing biofilm masses between different concentration of siderophore were determined by performing two-sample T-tests assuming equal variances.

### Optimization studies

#### Two level factorial designing.

Two level factorial design was adopted in prioritizing the constituents of the ISP2 medium to enhance siderophore production by the isolate following the procedure adopted by Murugappan et al. [17] with slight modifications. All the constituents of the ISP2 medium and hours of incubation were considered as independent variables. Influence of all the variables was tested at two levels, high (+1) and low (−1). The levels were chosen based on their actual proportion in the ISP2 medium as a central point. Influence of the variables on siderophore production was determined based on the t-values (P < 0.05.). Experiments were carried out in batch culture at 30°C for 10 days. Triplicates were maintained for all the experiments. Siderophore production was considered as the response on the influence of the variables at different levels.

Based on the 2 level factorial design, three main constituents (carbon, amino acid and nitrogen sources) of ISP4 medium viz., starch, casein and KNO_3_ with high t-values were selected for optimization studies. Influence of different concentrations of the three variables on siderophore production was analysed using response surface methodology (RSM) of central composite design (CCD) [34].

Twenty set of experiments were formulated using Design Expert software (Version 7.16 Stat-Ease Inc., Minneapolis, MN, USA) to determine the optimum concentration of the three variables (n = 3) at five levels (α = 2), while the other constituents in the medium were kept constant. The experiments were carried out in 3 L bioreactor (2-L working volume) following the steps briefed in the siderophore purification process. Second-order polynomial equation was employed to fit the experimental data using the following equation [35].


Yi =β0+ β1χ1 + β2χ2+ β3χ3+ β11χ21+ β22χ22+ β33χ23+ β12χ1χ2+ β13χ1χ3+ β23χ2χ3

where Yi is the predicted response; χ1, χ2, and χ3 are independent variables; β0 is the offset term; β1, β2, and β3 are the linear effects; β11, β22, and β33 are squared effects; and β12, β13, and β23 are the interaction term effects. The significance of the model equation and model terms were evaluated by F-test. The quality of the fit of the polynomial equation was expressed by coefficient of determination (R2). Contour plots and 3D surface graphs were generated to illustrate the interaction between the factors and the optimum value of each factor influencing siderophore production.

## Results and discussion

Two different *Streptomyces* *spp.* were isolated from the sea water. Of the two isolates, only one was found to produce siderophore. Change in the colour of the culture supernatant to dark yellow on addition of FeCl_3_ indicates the presence of siderophore. Appearance of orange colour in CAS liquid and formation of yellow hallow in CAS agar plate confirms siderophore production by the isolate. Siderophore mediated iron acquisition in marine *Streptomyces* sp was reported by Alam et al. [36]. Though siderophores are not essential for the growth or multiplication, they confer a competitive advantage to the host by confiscating iron from the environment [37]. Marine *Streptomyces spp.* were reported to produce 7600 bioactive secondary metabolites [38]. Identifying a potential strain is a prerequisite for discovering novel natural products and the one strain-many compounds (OSMAC) strategy is effective for discovering novel microbial metabolites [39]. *In lieu* with the findings of Chen et al. [40] *Streptomyces sp*. isolated from the marine surface water was found to produce siderophores during the stationary phase under iron limited conditions.

**Fig 1:**
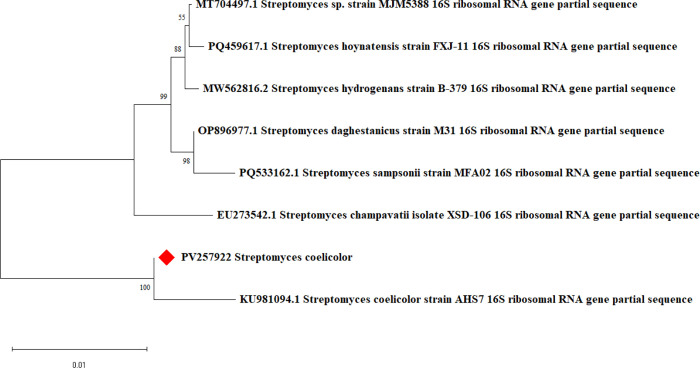
Phylogenetic tree depicting the evolutionary relationships of *S. coelicolor* based on 16S rRNA partial gene sequences comparison following Neighbor-Joining method.

Morphological, physiological, biochemical and phylogenetic analysis confirmed that the strain showed maximum identity towards *Streptomyces coliecolar*. Microscopic observation of the isolate revealed the presence of aerial coiled mycelia and arrangement of spores in chains. The micro-morphological characteristics like spore formation, dimorphic mycelia (aerial and substrate mycelium) and the Gram-positive nature revealed the possibility of the isolate to the genus *Streptomyces*. The macroscopic appearance of isolate showed white powdery colonies in the ISP2 medium and prominent red colour pigmentation on the bottom of the colony. The isolate was able to survive and grow in alkaline conditions (resistance to 20% NaCl) representing the halophilic nature The biochemical tests results indicate that the isolate was capable of hydrolysing starch, gelatin and casein (Table 1). The ideal temperature for the growth was found to be 28 and 30°C and are able to grow even at 40°C. Equally, similar biochemical and physiological characteristics were reported for several species of *Streptomyces* by Undabarrena et al. [41]. Comparison of 16S rRNA gene sequence of the isolate with the nucleotide sequence database revealed 100%sequence similarity with that of the genus *Streptomyces*. The 16S rRNA sequence of the isolate was submitted to Genbank (PV257922). Further, phylogenetic tree construction using neighbor-joining method revealed that the isolate was closely related to *Streptomyces coelicolor* (Fig 1)*.*

**Table 1:** Morphological, physiological and biochemical characters of *Streptomyces coelicolor.*

Morphological Characteristics	Results
Cultural characteristic	
Growth	+++
Colour of substrate mycelium	Dark brown
Colour of aerial mycelium	Brown
Soluble pigments produced	yellow
**Biochemical Characteristics**	
Degradation of Starch	+
Degradation of Casein	+
Degradation of Gelatin	+
Degradation of Glucose	+
Degradation of Lactose	+
Degradation of Sucrose	+
Degradation of Citrate	+
H2S production	+
Production of Urease	+
Mannitol	+
Catalase	+
**Physiological characteristic**	
Gram staining	G^+ve^
Effect of Temperature	
15°C	–
25°C	+ +
30°C	+ + +
40°C	+
50°C	–
Effect of pH	
5	–
6	–
7	+
8	+ + +
9	++
Effect of NaCl	
1%	++
3%	+ + +
6%	++
9%	+
12%	–

(G^+ve^): Gram positive; (-): Absence of growth; (+): Indicates the growth and number of (+) indicates the level of response of bacterial growth.

Marine *Streptomyces spp.* were chosen for this study for two reasons: firstly, they remain largely unexplored, secondly, they serve as a repository of a diverse and valuable natural products with significant biological activities [42]. In the recent years, the number of novel secondary metabolites discovered from marine *Streptomyces* has surpassed the number from their terrestrial counterparts [43].

### Characterization of siderophores

**Fig 2:**
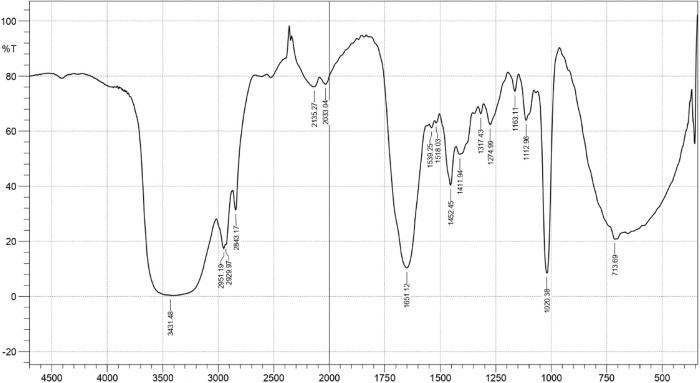
FT-IR spectrum illustrating the functional groups present in the siderophore isolated from *S. coelicolor.*

Siderophore produced by the isolate was confirmed as hydroxomate type based on Tetrazolium test and spectrophotometric assay (peak at 409 nm). Further characterization, illustrate the dihydroxomate nature based on the broad shift in λ max (52 nm) at different pH (4–9) and the number of bonds the ligand formed with metal ions was found to be hexadentate based on the retention of red colour at a wide pH (4–9) range (Table 2). Sarvepalli et al. [44] reported hydroxamate type siderophore production in *S. acidiscabies*. Liu et al. [45] reported the synthesis of 2,3-dihydroxybenzoate (DHB) moiety with structural variations in *S. varsoviensis*. Although the above analytical techniques determine the nature of the siderophore produced, they fail to give structural information and can be easily misinterpreted. In contrast, FT-IR, NMR and GC-MS allow for detailed structure analysis. In the present study, IR spectrum of the siderophore after purification process showed a broad peak at 3,431.48 cm^-1^, indicating the presence of the N–H moiety. A strong peak at 1651.12 cm^-1^ indicates C = O stretching. Peak at 1020.38 indicates C-N stretching. A band at 1452.45 cm^-1^ is due to the C-H bending vibrations. IR spectrum provides evidence for the possible presence of aromatic ring in the siderophore (2136.27 cm^-1^ represents C = C stretching in the aromatic ring). The IR spectral result of this study is in accordance with Siebner – Freibach et al. [46]. The peaks at 2951.19 cm^-1^ and 2929.97 cm^-1^ may be due to the bending vibration of methylene moiety. The lowering of the C = O stretch peak may be due to conjugation as well as intramolecular hydrogen bonding (Fig 2). Overall, the FT-IR spectrum indicates the hydroxamate type of siderophore.

**Table 2:** Determination of the ligand denticity of hydroxamate siderophore produced by *S. coleicolor.*

λ max (nm) of ferrate siderophore		λ max (nm)	Colour of the ferrate Siderophores in electrophoresis	Inference	Colour of the ferrate hydroxamate	Binding properties
pH	λ max (nm)					
4	405	8	Red	Trihydroxamate	Red	Hexadentate
5	407					
6	400					
7	406					
8	409					
9	405					

**Fig 3:**
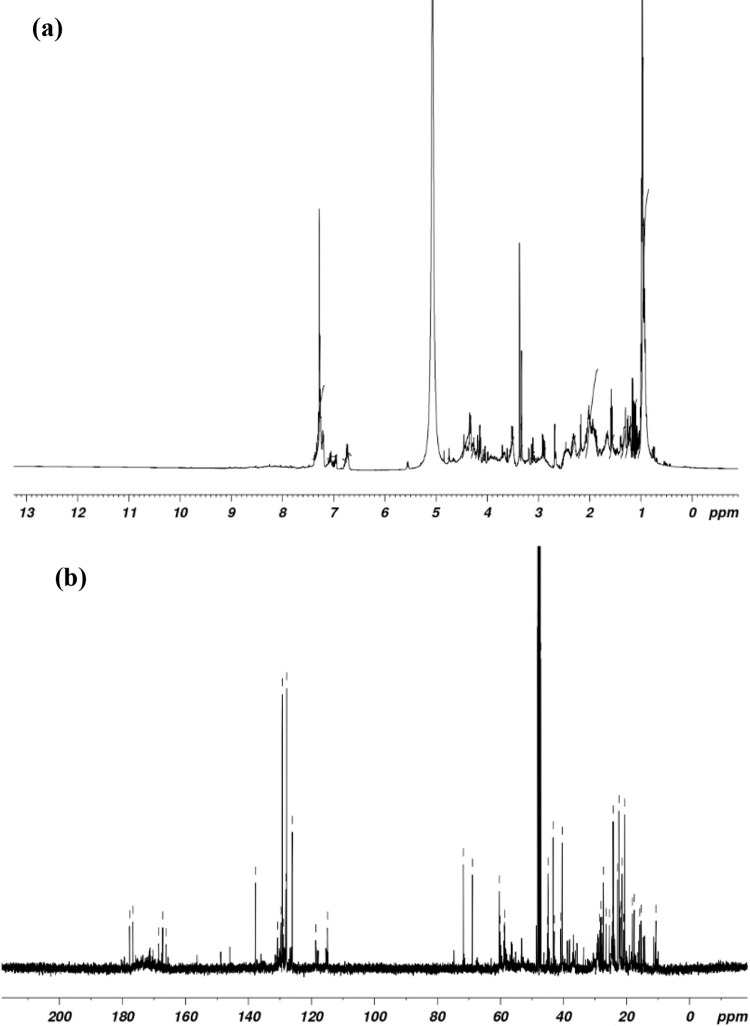
^1^H NMR spectrum (a) displays the characteristics proton signals and ^13^C NMR spectrum (b) reveals distinct carbon resonance of the siderophore produced by *S. coelicolor.*

Proton NMR spectrum of hydroxamate siderophore (Fig 3a) exhibit three triplet peaks at 1.24, 1.51, and 2.18 ppm that represent the presence of CH_2_-CH_2_, CH_2_-CH_2_ and CH_2_ = CH respectively. A doublet peak at 1.47 ppm indicates CH_2_ = CH, singlet peaks at 0.95 and 3.57 ppm may be due to =CH and aliphatic OH (hydroxyl) group respectively. A peak at 5.23 and 7.51 ppm revealed the presence of aromatic ring. The resonance at 3.50 ppm was referred as solvent peak. The ^1^H NMR spectrum establishes the possible presence of trihydroxamate type of siderophore.

^13^C NMR spectrum of siderophore produced by *Streptomyces coelicolor* showed three peaks at 130.01, 138.19, 144.98 ppm indicating the presence of carbonyl carbon (Fig 3b). The peaks in the region 126.44 to 128.63 and 17.19 to 72.29 ppm may be due to the presence of aromatic carbon and methyl carbon group respectively. The other peaks at 27.33, 28.48 and 31.67 may be due to the presence of methyl group. Comparison of the NMR spectra associated with each analyte peak to a library of known analytes identified the presence of hydroxamate siderophores or their precursors and breakdown products [47,48].

**Fig 4:**
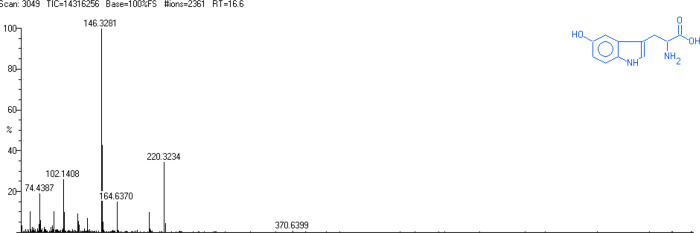
GC-MS analysis of the siderophore produced by *S. coelicolor.* Prominent peaks with their corresponding mass spectra supporting its structural characterization.

Mass spectrometry results illustrate that the siderophore secreted from the *Streptomyces* has a mass to charge ratio (m/z) of 220 (Fig 4). GC- MS analysis further supports that the *Streptomyces coelicolor* produce a hydroxamate moiety. Since one of the fragments has the typical mass of a derivatized aromatic group. Interpreting with the available literatures, the results obtained in this study allow the postulation of the siderophore produced by the *Streptomyces coelicolor* as ferrioxamine type [40].

Binding potential of siderophore with different divalent metals was visualized by the change in the colour of the CAS medium. Based on the change in the colour of the CAS agar it can be illustrated that the order of complexation of siderophore with the metals was found to be Fe > Zn > Co = Cd > Mn > Pb. Results of the siderophore metal complexation efficiency in microtiter plate assay falls in line with that of CAS agar test. Results obtained in microtiter plate coincide with that of CAS agar plate. The results of both the tests confirm that the hydroxamate siderophore produced by *Streptomyces coelicolor* was found to bind with most of the divalent metals tested, except chromium and aluminium (Table 3). Nair et al*.* [28] reported that the binding intensity of siderophore differs with different metals. Bi et al. [49] revealed the binding of siderophores with actinides and divalent cations such as magnesium, manganese, zinc, copper, nickel, gallium and radionuclides such as plutonium. Patel et al. [50] reported that siderophore produced by *Pseudomonas aeruginosa* RZS3 and *Alcaligenes sp.* RZS2 bind to nickel (Ni) and mercury (Hg). Yarnell and Washington [51] reported binding of siderophore with toxic metals like lead and cadmium. But still there is much to be explored concerning the role of environmental factors and the economic feasibility of employing microbial siderophores for metal recovery from the environment.

**Table 3:** Determination of siderophore-metal complexation.

Metals	Siderophore produced by the isolate *Streptomyces coelicolor*	
	CAS assay	Zone (mm)
Zn	+	1.20 ± 0.02
Cu	–	0
Co	+	1.19 ± 0.01
Fe	+	1.23 ± 0.03
Cd	+	1.19 ± 0.02
Al	–	0
Pb	+	0.41 ± 0.02
Mn	+	1.17 ± 0.02

Results were shown as average of three replicates (n = 3) ± standard deviation.

(+) – indicates positive reaction; (-) – indicates negative reaction.

**Fig 5:**
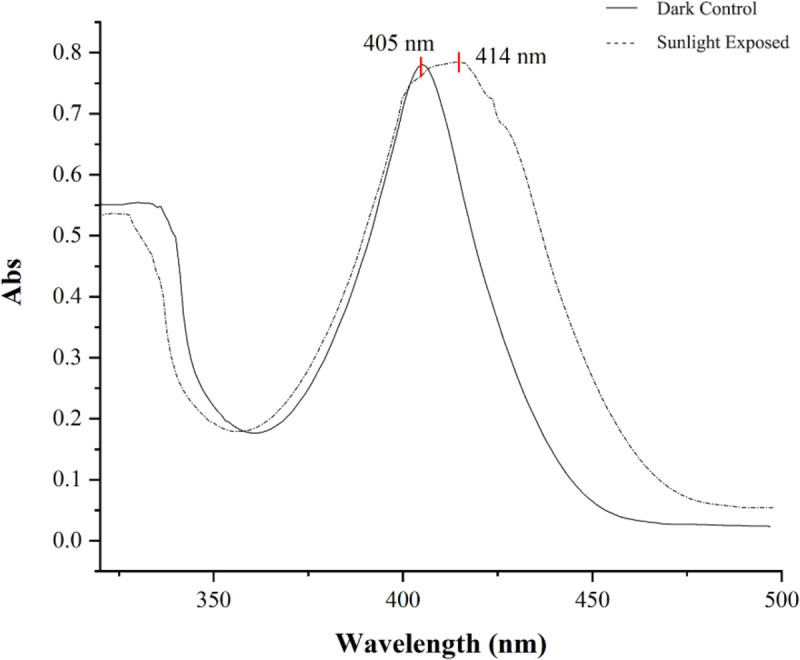
The siderophore produced by *S. coelicolor* exhibits photoreactive properties, showing a slight shift in its λ max upon exposure to sunlight.

A narrow shift in the λ max (405–414 nm) was observed in the UV visible spectrum on exposure of the siderophore to sun light (Fig 5). The shift in the λ max is due to the photo reactivity of the siderophore. Barbeau et al. [52] reported photolytic induced transfer of charge from ligand to metal resulting in the oxidation of ferric (Fe^3^+ ) to ferrous (Fe^2^+ ) state.

**Fig 6:**
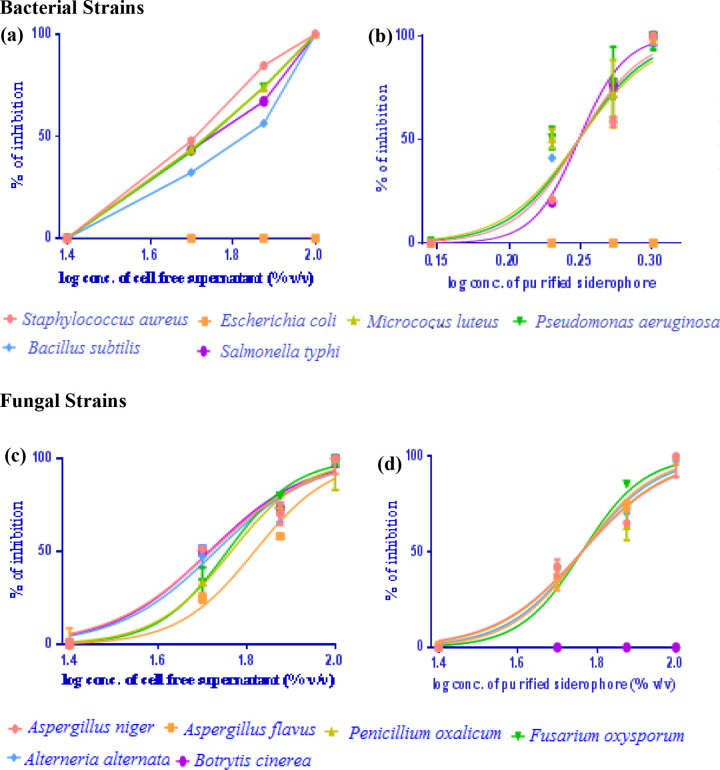
Dose–response curve depicting the concentration-dependent antimicrobial (a&b) and antifungal (c & d) activity of the cell free supernatant (a & c) and the siderophore (b & d) produced by *S. coelicolor* illustrating the comparative efficacy of the crude extract and the siderophore.

**Fig 7:**
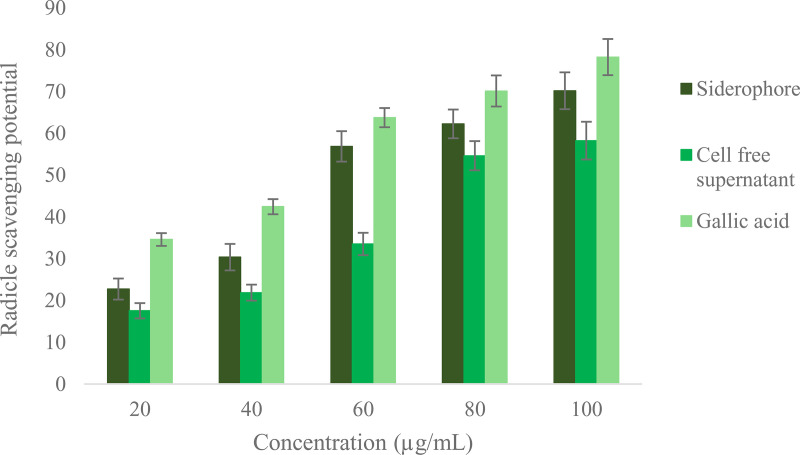
Antioxidant potential of the siderophore and cell free supernantant of *S. coelicolor* along with the standard (Gallic acid) following DPPH assay.

Dose–response curves obtained by plotting the different concentrations of the siderophore, cell free supernatant against bacterial and fungal pathogens are shown in Fig 6. High R^2^ values (equal to 1) obtained in the regression plot represent the inhibitory efficiency of the siderophore and cell free supernatant of *S. coelicolor* towards bacterial and fungal pathogens. The percentage inhibition model provides a good linearization of the dose-response data for the tested pathogens. From the dose response analysis, it can be deduced that the cell free supernatant of the isolate *Streptomyces coelicolor* is more effective than the siderophore extracted (Fig 7). Of the different bacteria and fungi tested, *E. coli* was found to be resistant, *Staphylococcus aureus* and *Penicillium oxalicum* was found to be more susceptible. Secondary metabolites derived from *Streptomyces* were reported to exert antifungal activity by Chevrette et al. [53]. Siddharth et al. [54] reported the antagonistic activity of cell free supernatant of marine *Streptomyces sp*. against bacterial and fungal pathogens.

Apart from iron sequestration, siderophores exhibit concentration dependant free radical scavenging activity, contributing to the protection of cells from oxidative stress. Based on the IC_50_ values and the range of antioxidant activities it can be deduced that siderophore exhibit greater free radical scavenging potential (51.23 µg/mL) than the cell free supernatant of *S. colicolar* (64.14 µg/mL). Results were compared with standard gallic acid (49.02 µg/mL) (Fig 7). It is evident from the results that the secondary metabolites especially siderophores produced by *S. coelicolor* might be responsible for the antioxidant activity. Siderophores, protochelin and azotochelin produced by *Azotobacter vinelandii* were reported to prevent oxidative damage catalysed by superoxide and Fe^3+^ [55]. Schrettl et al. [56] linked siderophore synthesis to oxidative stress resistance in the fungus *Aspergillus fumigatus.*

**Fig 8:**
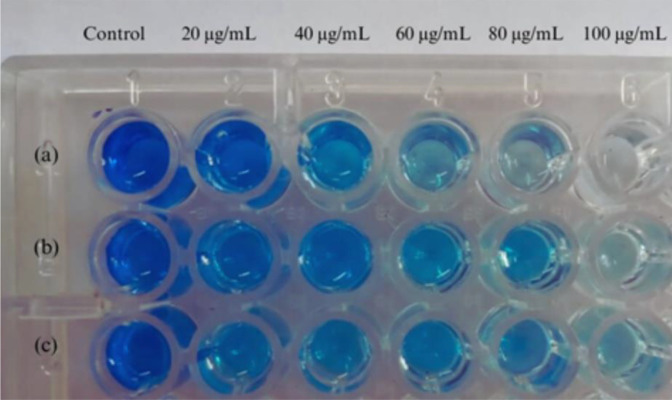
Inhibition of violacein pigment production in *Chromobacterium violaceum* by (a) Desferrioxamine (b) Cell Free Supernatant (c) Purified siderophore demonstrating their quorum quenching activity.

It is evident from the results that siderophore produced by *S. coelicolor* exhibit quorum quenching potential (Fig 8) and effectively prevent biofilm forming efficiency (Table 4) of the pathogens. Biofilm inhibition and quorum quenching assays were carried out in this study because the above mechanisms protect the microbes against chemical inhibitors and prevent interspecies communication respectively [57]. Therefore, co-culturing siderophore producing organisms with pathogens prevent signalling molecules mediated communication and coordination among microorganisms. Khasheii et al. [4] reported that siderophore act as a signalling molecule in regulating the interactions between bacterial cells and inhibit the expression of virulence factor in pathogenic bacteria. Bioactive compounds from Streptomyces *spp.* were reported to be effective in inhibiting biofilm formation in *Streptococcus aureus* [58]. Secondary metabolites of *S. colifornicus* were reported to inhibit biofilm formation in *Staphylococcus aureus* [59]. *Streptomyces sp.* isolated from the marine sediments were reported to inhibit biofilm formation in *E. coli* and *S. aureus* [60].

**Table 4:** Biofilm inhibition potential of the purified siderophore and cell free supernatant of *S. coelicolor.*

	Siderophore	Cell free supernatant	Desferrioxamine
20 µg/mL	1.57 ± 0.17	1.87 ± 0.21	1.38 ± 0.14
40 µg/mL	1.50 ± 0.12	1.66 ± 0.18	1.21 ± 0.13
60 µg/mL	1.24 ± 0.16	1.59 ± 0.17	0.98 ± 0.06
80 µg/mL	1.12 ± 0.08	1.43 ± 0.13	0.86 ± 0.08
100 µg/mL	0.96 ± 0.10	1.33 ± 0.09	0.77 ± 0.11

**Fig 9:**
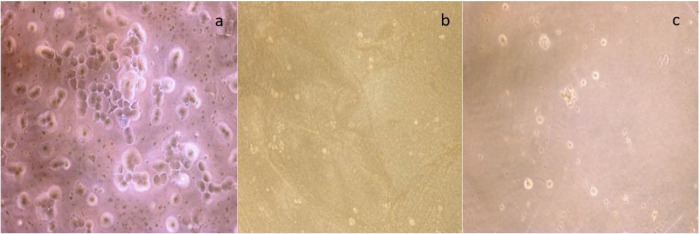
Microphotographs of MCF7 cancer cells. (a) untreated cells exhibiting normal morphology and high cell density; (b) cells exposed to the IC₅₀ concentration of purified siderophore; and (c) cells treated with the commercially available siderophore, desferrioxamine. Both treatments show decreased cell density and distinct morphological alterations characteristic of cell death.

The cytotoxic activity of commercial siderophore (desferrioxamine) and siderophore produced by *S. coelicolor* was determined using MTT assay. The proliferative effect of MCF-7 cells was confirmed by cell morphology evaluation by inverted fluorescent microscopy (Fig 9). On siderophore treatment cells get condensed and shrank. Most of the cells get detached from the plates and remain floating in aggregates. Apart from morphological analysis, tumor cell inhibition was measured to confirm the effects of siderophore treatment. Tumour cells (MCF-7) showed significant decrease in cell proliferation on treatment with commercial and microbe derived siderophores (Fig 9).

The IC_50_ value of desferrioxamine and siderophore produced by *S. coelicolor* was 671.52 and 177.87 μg/mL respectively. To validate the experimental studies, viability of normal cells was also checked. Interestingly, inhibition of normal cells on siderophore treatment was comparatively lesser with that of cancer cells (Results not shown). From this it can be concluded that siderophores produced by the Streptomyces *spp.* have fewer side effects. These results suggested the beneficial effect of siderophores in normal cells with less cytotoxicity than conventional anti-cancer drug while maintains its anti-tumor effect in cancerous cell inducing apoptotic activity. Saha et al. [61] illustrated that enterobactin, an iron chelating bacterial siderophore, arrests cancer cell proliferation without harming normal epithelial cells. Gokarn et al. [62] and Pita-Grisanti et al. [2] reported that siderophore can be used as an adjunct along with the drug in chemotherapy to inhibit tumour cells.

**Fig 10:**
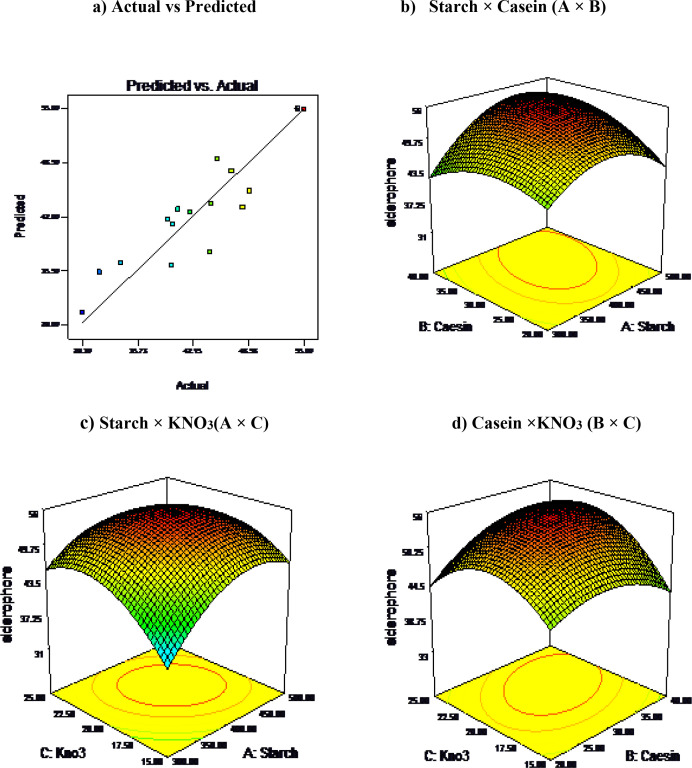
Three-dimensional response surface plot showing the effect of different variables on siderophore production in *S. coelicolor.*


Results of central composite design on siderophore production in *S. coelicolor* with the predicted and observed responses are presented (Fig 10a). The second-order polynomial equation has been used for the stepwise regression analysis to estimate the effect of the three variables on siderophore production. The main, interaction and quadratic effects of variables are represented by the second order polynomial equation as:

$$\begin{array}{c} \text{Siderophore}\;\%\;\text{units}=\;\text{54}.\text{90}+\text{2}.\text{43}\;\times x\text{1}+\text{1}.\text{36}\;\times x\text{2}+\text{1}.\text{80}\;\times x\text{3}-\text{5}.\text{47}\times x{\text{1}}^{\text{2}}-\text{3}.\text{44}\\ \;\;\;\;\;\;\;\;\;\;\times \;x{\text{2}}^{\text{2}}-\text{5}.\text{46}x{\text{3}}^{\text{2}}+\text{2}.\text{69}\times x\text{1}x\text{2}-\text{2}.\text{19}\times x\text{1}x\text{3}-\text{2}.\text{34}\times x\text{2}x\text{3} \end{array}$$

The statistical significance of the model equation was evaluated by analysis of variance (ANOVA), which showed that the regression is statistically significant at 99% (P < 0.05) confidence level (Table 5). The analysis of variance for the three variables indicated that siderophore production can be well described by a polynomial model with a relatively high co-efficient of determination. The correlation coefficient (R_2_) value of 0.8895 indicates that the above model is well fitted to the experimental data. In addition, the adj. R_2_ value of 0.7900 is also closer to R_2_ value and suggests the good agreement of model prediction with experimental data. The values of F statistic was found to be 8.94 with (P < 0.001) respectively. This implies that the model is significant. The response surface plots are graphical representation of the regression equation and the interaction between the variable can be inferred from the shape of the surface plots. Influence of interaction between starch and casein, starch and KNO_3_, casein and KNO_3_ on siderophore production was presented (Fig 10 b, c and d). It is evident from the figures that the interactive effect of starch and caesin had a significant effect on siderophore production.

**Table 5:** Analysis of variance (ANOVA) for the polynomial model of siderophore production.

Source	SS	Df	MS	F-value	Prob (P) > F
Siderophore production: [R^2^ = 0.8895, adj R^2^ = 0.7900, pred R^2^ = 0.0507, CV = 8.43%]					
Model	1161.40	9.00	129.04	8.94	0.0010
A-Starch	25.13	1.00	25.13	1.74	0.2164
B-Casein	25.13	1.00	25.13	1.74	0.2164
C-KNO3	44.15	1.00	44.15	3.06	0.1109
A × B	57.78	1.00	57.78	4.00	0.0733
A × C	38.28	1.00	38.28	2.65	0.1345
B × C	43.71	1.00	43.71	3.03	0.1124
A^2^	431.91	1.00	431.91	29.92	0.0003
B^2^	170.70	1.00	170.70	11.83	0.0063
C^2^	429.13	1.00	429.13	29.73	0.0003
Residual	144.34	10.00	14.43		
Lack of fit	144.34	5.00	28.87		
Pure Error	0.000	5.00	0.00		
Total	1305.74	19.00			

SS-sum of squares; df-degrees of freedom; MS-mean squares.

## Conclusion

To conclude, siderophores represent very exciting family of metal chelators that have extremely broad spectrum of biological activities. The results of this study provide a “proof of concept” that siderophore mediated iron chelation have the capability to function as antimicrobial, antioxidative and cytotoxic agents. Furthermore, it is evident from the study that siderophores not only chelate iron but also have the potential to bind with other bivalent metal ions facilitating bioremediation. Future research directions have to focus on the comprehensive pharmacological investigations ought to assess the safety and effectiveness of the isolated compounds, bringing us one step closer to possible clinical trials and contributing to the resolution of the long-standing challenge of antibiotic resistance. Furthermore, *in vivo* studies are in progress to validate the efficacy, pharmacokinetics, and safety of the siderophore in living systems.

## Supporting information

S1 Table Antimicrobial activity of cell free supernatant and purified siderophore produced by *Streptomyces coelicolor.* (PDF)

S2 Table Antioxidant potential of the siderophore and cell free supernantant of *S. coelicolor* along with the standard (Gallic acid) following DPPH assay. (PDF)

S3 Table Central composite design (CCD) matrix with experimental and predicted values of siderophore production in *S. coelicolor.* (PDF)
