# Supplementary material for: Optimization, characterization and biological activity of siderophore produced by marine Streptomyces coelicolor
Source: PLoS One. 2026 Feb 2;21(2):e0341555. doi: 10.1371/journal.pone.0341555 (PMC12863511; doi:10.1371/journal.pone.0341555)
Supplement: S1 Table — (PDF) [file pone.0341555.s001.pdf]

S1 Table. Antimicrobial activity of cell free supernatant and purified siderophore produced by *Streptomyces coelicolor*.

Bacterial and Fungal isolates	Zone of inhibition (mm)							
	Cell free supernatant (%)				Purified siderophore (%)			
	25	50	75	100	25	50	75	100
Staphylococcus aureus (MTCC 2940)	14.44 (±0.16)	15.22 (±0.22)	16.88 (±0.50)	17.58 (± 0.53)	11.15 (±0.36)	12.44 (±0.21)	13.55 (±0.24)	14.62 (± 0.64)
Escherichia coli (MTCC 739)	0.00	0.00	0.00	0.00	0.00	0.00	0.00	0.00
Micrococcusluteus (MTCC 106)	11.67 (±0.41)	13.31 (±0.38)	14.23 (±0.27)	15.12 (± 0.77)	9.96 (±0.55)	10.33 (±0.26)	11.89 (±0.35)	12.24 (± 0.54)
Pseudomonas aeruginosa (MTCC 424)	7.72 (±0.38)	8.54 (±0.53)	9.27 (±0.61)	10.66 (± 0.57)	5.21 (±0.17)	5.53 (±0.26)	6.87 (±0.45)	7.14 (± 0.27)
Bacillus subtilis (MTCC 1427)	8.32 (±0.31)	11.91 (±0.35)	12.54 (±0.21)	13.57 (± 0.44)	8.12 (±0.	9.56 (±0.17)	10.45 (±0.46)	11.69 (± 0.56)
Salmonella typhi (MTCC 733)	5.56 (±0.31)	6.97 (±0.43)	7.01 (±0.52)	7.73 (± 0.61)	2.89 (±0.15)	3.76 (±0.22)	4.88 (±0.31)	5.64 (± 0.52)
Aspergillus niger (NCIM 586)	10.67 (±0.42)	12.63 (±0.53)	14.87 (±0.62)	15.85 (± 0.40)	12.25 (±0.31)	13.21 (±0.26)	14.67 (±0.25)	14.32 (± 0.15)
Aspergillus flavus (MTCC 7133)	10.88 (±0.51)	12.95 (±0.43)	13.21 (±0.47)	14.43 (± 0.26)	8.15 (±0.43)	9.55 (±0.41)	10.21 (±0.26)	11.28 (± 0.34)
Penicillium oxalicum (MTCC 4931)	14.81 (±0.22)	16.32 (±0.52)	17.23 (±0.65)	18.91 (± 0.53)	11.83 (±0.41)	12.56 (±0.13)	13.25 (±0.41)	15.44 (± 0.32)
Fusarium oxysporum (MTCC 4894)	12.43 (±0.27)	14.52 (±0.31)	15.32 (±0.41)	16.25 (± 0.34)	10.11 (±0.38)	11.16 (±0.32)	12.75 (±0.31)	13.11 (± 0.42)
Alterneria alternata (MTCC 9617)	13.88 (±0.19)	15.21 (±0.15)	16.55 (±0.54)	17.24 (± 0.49)	11.31 (±0.33)	12.26 (±0.25)	13.41 (±0.42)	14.78 (± 0.35)
Botrytis cinerea (MTCC 2175)	7.72 (±0.31)	8.11 (±0.36)	9.34 (±0.26)	10.32 (± 0.21)	0.00	0.00	0.00	0.00

Results were shown as average of three replicates (n=3) ± standard deviation
