# Supplementary material for: Optimization, characterization and biological activity of siderophore produced by marine Streptomyces coelicolor
Source: PLoS One. 2026 Feb 2;21(2):e0341555. doi: 10.1371/journal.pone.0341555 (PMC12863511; doi:10.1371/journal.pone.0341555)
Supplement: S2 Table — (PDF) [file pone.0341555.s002.pdf]

S2 Table: Antioxidant potential of the siderophore and cell free supernantant of *S. coelicolor* along with the standard (Gallic acid) following DPPH assay.

**Concentration (µg/mL)**	**Siderophore**	**Cell free supernatant**	**Gallic acid**
20	22.73±1.53	17.53±1.83	34.58±2.52
40	30.35±1.81	21.87±1.91	42.43±3.17
60	56.86±2.3	33.52±2.67	63.74±3.64
80	62.24±3.72	54.63±3.49	70.12±3.44
100	70.17±4.33	58.24±4.52	78.23±4.41
