# Supplementary material for: Optimization, characterization and biological activity of siderophore produced by marine Streptomyces coelicolor
Source: PLoS One. 2026 Feb 2;21(2):e0341555. doi: 10.1371/journal.pone.0341555 (PMC12863511; doi:10.1371/journal.pone.0341555)
Supplement: S3 Table — (PDF) [file pone.0341555.s003.pdf]

**S3 Table. Central composite design (CCD) matrix with experimental and predicted values of siderophore production in *S. coelicolor*.**

| Run | Starch | Casein | $KNO_3$ | Siderophore Units (%) | |
|---|---|---|---|---|---|
| | | | | Experimental | Predicted |
| 1 | 500.00 | 40.00 | 25.00 | 48.70 | 45.05 |
| 2 | 300.00 | 40.00 | 25.00 | 55.00 | 54.90 |
| 3 | 400.00 | 30.00 | 20.00 | 33.80 | 36.44 |
| 4 | 400.00 | 13.18 | 20.00 | 31.30 | 35.33 |
| 5 | 400.00 | 46.82 | 20.00 | 44.10 | 37.78 |
| 6 | 231.82 | 30.00 | 20.00 | 40.40 | 42.88 |
| 7 | 300.00 | 40.00 | 15.00 | 39.70 | 36.19 |
| 8 | 300.00 | 40.00 | 15.00 | 45.00 | 48.95 |
| 9 | 400.00 | 30.00 | 28.41 | 39.20 | 41.64 |
| 10 | 500.00 | 20.00 | 15.00 | 39.80 | 41.08 |
| 11 | 568.18 | 30.00 | 20.00 | 46.60 | 47.45 |
| 12 | 300.00 | 20.00 | 25.00 | 47.90 | 43.09 |
| 13 | 400.00 | 30.00 | 11.59 | 29.30 | 30.45 |
| 14 | 500.00 | 20.00 | 25.00 | 41.80 | 42.49 |
| 15 | 500.00 | 40.00 | 15.00 | 44.20 | 43.50 |
